# Surfactant Interactions with Protein-Coated Surfaces: Comparison between Colloidal and Macroscopically Flat Surfaces

**DOI:** 10.3390/biomimetics5030031

**Published:** 2020-07-01

**Authors:** Helena Mateos, Alessandra Valentini, Francesco Lopez, Gerardo Palazzo

**Affiliations:** 1 CSGI (Center for Colloid and Surface Science), via Orabona 4, 70125 Bari, Italy; 2 School of Chemical Engineering, University of Birmingham, Birmingham B15 2TT, UK; valentini.a.1@pg.com; 3 Department of Agricultural, Environmental and Food Sciences (DiAAA) and CSGI, University of Molise, Via De Sanctis, 86100 Campobasso, Italy; lopez@unimol.it; 4 Department of Chemistry, University of Bari, via Orabona 4, 70125 Bari, Italy; gerardo.palazzo@uniba.it

**Keywords:** protein-coated surfaces, surfactant, DLS, ζ potential, BSA, colloids

## Abstract

Surface interactions with polymers or proteins are extensively studied in a range of industrial and biomedical applications to control surface modification, cleaning, or biofilm formation. In this study we compare surfactant interactions with protein-coated silica surfaces differing in the degree of curvature (macroscopically flat and colloidal nanometric spheres). The interaction with a flat surface was probed by means of surface plasmon resonance (SPR) while dynamic light scattering (DLS) was used to study the interaction with colloidal SiO_2_ (radius 15 nm). First, the adsorption of bovine serum albumin (BSA) with both SiO_2_ surfaces to create a monolayer of coating protein was studied. Subsequently, the interaction of these BSA-coated surfaces with a non-ionic surfactant (a decanol ethoxylated with an average number of eight ethoxy groups) was investigated. A fair comparison between the results obtained by these two techniques on different geometries required the correction of SPR data for bound water and DLS results for particle curvature. Thus, the treated data have excellent quantitative agreement independently of the geometry of the surface suggesting the formation of multilayers of C_10_PEG over the protein coating. The results also show a marked different affinity of the surfactant towards BSA when the protein is deposited on a flat surface or individually dissolved in solution.

## 1. Introduction

Protein/surfactant interactions find important applications in industry and science. Their behavior at the air/liquid or liquid/liquid interface has been widely investigated due to their importance in the formation and stabilization of foams and emulsions [1,2,3,4,5,6,7]. Protein adsorption to solid surfaces is also related to dirt cleaning, or ultimately, it can be associated with biofilm formation, in which case, it is important to minimize or remove proteins adsorbed to the solid surface [8,9,10]. Studies of adsorption and protein/surfactant interaction at the liquid/solid interface are, however, not common. Furthermore, predicting protein/surfactant interactions at a solid surface is often difficult and not intuitive, especially when dealing with soft and flexible proteins that might change conformation when in contact with a surface. The subject of this paper is to investigate the potential use of colloidal surfaces instead of flat macromolecular ones for the research of interactions at the liquid/solid interface. The use of colloidal surfaces in combination with dynamic light scattering (DLS) could present many potential advantages since it can be applied to a wide range of materials including those that are unsuited for the external coatings. Furthermore, working with colloidal solutions increases substantially the analyzed surface area, which could lead to more representative results.

**Scheme 1:**
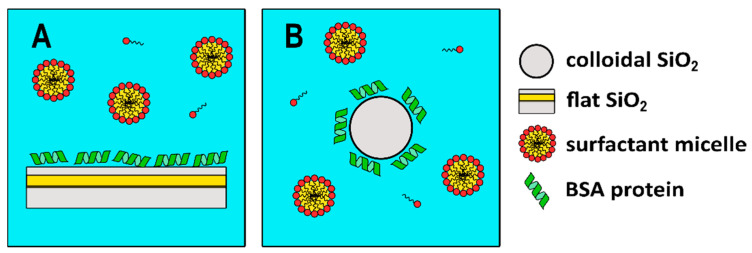
Graphical representation of the interaction between bovine serum albumin (BSA)-coated SiO_2_ and surfactant on both flat (**A**) and colloidal surfaces (**B**).

We have studied the interaction between bovine serum albumin (BSA) and the non-ionic surfactant C_10_PEG, at the glass/water interface in flat and colloidal forms (Scheme 1). Using glass is an interesting case scenario since it is a very common household surface. On the other hand, BSA’s adsorption at a variety of interfaces has been extensively studied previously [10,11,12,13,14,15] and C_10_PEG is a common and relevant surfactant widely used in industry. In this paper, we compare the results of the interactions between both solid-surface conformations using surface plasmon resonance (SPR), dynamic light scattering (DLS), and laser Doppler electrophoresis (LDE) measurements.

## 2. Materials and Methods

Bovine serum albumin (BSA) protein (purity > 98%) and Ludox AM colloidal silica system were purchased from Sigma-Aldrich (St. Louis, MO, USA) and used without any further purification. Na_2_HPO_4_ and NaH_2_PO_4_ were purchased from Fluka (Buchs, Switzerland) and J. T. Baker (Phillipsburg, NJ, USA), respectively. The water was purified by means of a Milli-Q system (Millipore Corp., Bedford, MA, USA) and hereafter is denoted as MQ water. Aqueous phosphate buffer was prepared from MQ water. The surfactant (decanol ethoxylated with an average number of eight ethoxy (EO) groups) was supplied by Sasol with a purity grade of 95%.

We used surface plasmon resonance (SPR) to evaluate the interaction of C_10_PEG on a previously deposited BSA layer on a flat, macroscopic silica-coated sensor surface (the thickness of the silica layer was 10 nm). A dual-wavelength (670 and 785 nm), multiparametric SPR instrument (MP–SPR Navi 200, BioNavis Ltd., Tampere, Finland) was used to record the SPR spectra. Contrary to traditional SPR, MP SPR allows the measurement up to 5 μm above the solid surface. Spectra were acquired in the range of 50° to 77.5°. The fluidic system was cleaned before and after each experiment using a glass slide in the place of the sensor. A flow of 200 μL/min of purified MQ water was set for 10 min followed by a 1% SDS solution in water for 10 min and the final flow of MQ water for other 10 min. BSA and surfactant samples were prepared in phosphate-buffered saline (PBS) (pH 7.2) and injected (250 μL injection loops) on a silica-coated sensor in kinetic titration mode at a constant flow of 20 μL/min. All the experiments were done in triplicate. The affinity constants were estimated using Winspall 3.02 (Max Planck Institute for Polymer Research, Mainz, Germany) and OriginPro (OriginLab Corporation, Northampton, MA, USA) software.

The interaction between colloidal particles and BSA was studied by adding increasing BSA concentrations ranging from 0.08 to 8 mg/mL to a 2.5 mg/mL Ludox solution in 10 mM phosphate buffer at pH = 7.4. The resulting samples were analyzed by following the changes in ζ-potential (laser Doppler electrophoresis, LDE) and size distribution (dynamic light scattering, DLS) using a Nanosizer ZS (Malvern instruments, Malvern, UK). Instrumental conditions are explained elsewhere (see [16] for LDE and [17] for DLS. The ζ-potential was subsequently evaluated from the electrophoretic mobility according to the Smoluchowski approximation.

To study the interactions between BSA-coated Ludox particles and surfactant C_10_PEG_,_ we chose a solution of 2.5 mg/mL Ludox and 8 mg/mL BSA concentration. Then, surfactant was added in a 0.5 to 10 wt% concentration range keeping the buffer concentration constant. 

^1^H diffusion NMR and fluorescence measurements were performed using the same set-ups described elsewhere [17].

## 3. Results and Discussion

The experimental strategy used in this investigation is as follows: from SPR experiments it is possible to obtain information on the deposition of the analytes on a flat SiO_2_ surface. Then, DLS and EDL can be used to obtain the same information on a nano-sized SiO_2_ surface. The comparison of both sets of experiments allows us to compare the use of flat versus nanosized surfaces from the interaction of BSA and further interaction between BSA and a non-ionic C_10_PEG surfactant.

### 3.1. Interaction of BSA on a Silica Surface

**Figure 1:**
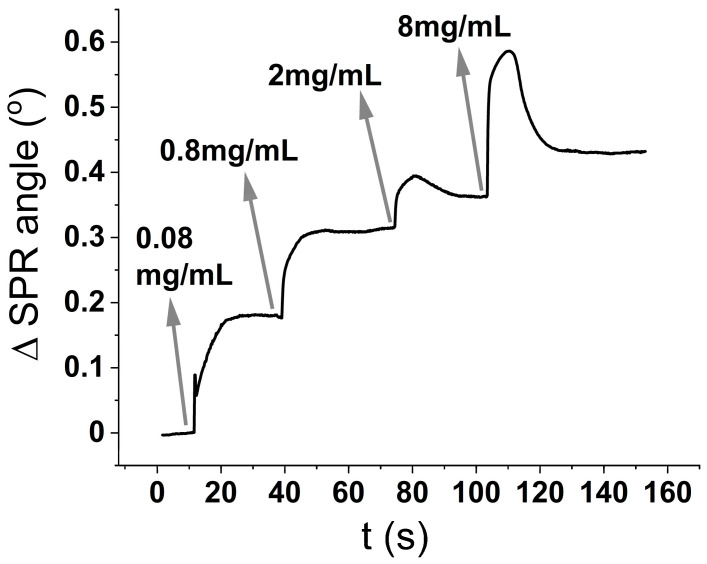
Surface plasmon resonance (SPR) sensogram of a kinetic titration of BSA on a macroscopic silica-coated sensor (the thickness of the silica layer was 10 nm).

A first analysis of the interaction between BSA and SiO_2_ is necessary to understand how to obtain a monolayer with a good surface coverage of the protein on the sensor chip’s surface. Moreover, understanding the deposition of BSA on the SiO_2_ surface is crucial to further investigate its interaction with surfactants. Therefore, we injected increasing concentrations of the protein to extract information about the affinity and needed concentration to create a good coverage on the chip. The experiment shown in Figure 1 is performed by sequential injections (also called kinetic titration) of the analyte (BSA) in increasing concentrations. After the first injection is finished, and only the buffer is being flushed, the SPR intensity signal reaches a stable plateau, meaning that the analyte is not being desorbed from the surface. Then, the second analyte concentration is injected, and so on. The change in the minimum angle with time can be related to either, an increase in the thickness of the deposited layer, or an increase in surface coverage.

**Figure 2:**
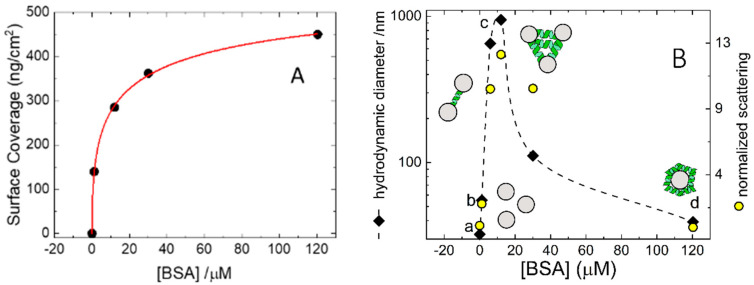
Interaction of BSA with a flat SiO_2_ surface. (**A**) Flat surface: calculated surface coverage from the SPR sensogram shown in Figure 1 fitted with Equations (4) and (6); best fit parameters are *K* = (6 ± 2) × 10^4^ M^−1^, *m_a_Q* = 620 ± 40 ng/cm^2^, *n* = 0.48 ± 0.03. (**B**) Curved colloidal surface: hydrodynamic diameters (left ordinate) and normalized scattering intensities (right ordinate) for SiO_2_ nanoparticles as a function of BSA concentration measured by means of dynamic light scattering (DLS). The curves correspond to: (**a**) ludox only, (**b**) [BSA] = 1.2 μM, (**c**) [BSA] = 12 μM, (**d**) [BSA] = 120 μM.

The surface coverage (ng/cm^2^) is usually calculated using the De Feiter equation(Equation (1)) [18], which predicts the surface coverage to be the optical thickness (*D_0_* in Equation (2)) divided by the refractive index increment with concentration (*dn/dC* Equation (3)):

(1) $$\Gamma =\frac{D_{0}}{dn/dC}$$

(2) $$D_{0}=d\left(n-n_{0}\right)$$

where *d* is the thickness of the deposited layer on the sensor, which was calculated by fitting the SPR curves at the plateau after each injection step using Winspall 3.02 and using 1.52 as the refractive index of BSA. The refractive index increment in Equation (1) can be calculated from a single wavelength measurement by knowing the shift in the total internal reflection (TIR) angle (${\Delta \theta }_{\mathrm{TIR}}$) caused by the change in the refractive index of the ligand bulk solution at a certain concentration *C_x_*.

(3) $$\frac{dn}{dC}=\frac{{\Delta \theta }_{\mathrm{TIR}}}{G\;C_{x}}$$

where *G* is a sensitivity factor for the change in the TIR angle with the change in the bulk solution refractive index. *G* is a constant for each measurement wavelength (86.3° for 670 nm, 87.5° for 785 nm). Since the calculated optical thickness increase (S) after each injection step (~0.002 nm) is significantly smaller than the size of a BSA molecule (~7 nm), the increase in the minimum angle after injections of BSA is solely related to an increase in the surface coverage and not to a formation of multiple layers. This result agrees with previous literature on the field, since the formation of negatively-charged BSA (−10 mV) monolayers onto negative silica surfaces (−50 mV) has been proven in the presence of PBS buffer and room temperature [12,14,15]. In the present case, the surface coverage increases upon exposure to increasing concentrations of BSA, and the surface coverage values can be plotted against analyte concentration obtaining the corresponding adsorption isotherm of Figure 2A. The adsorption isotherm relates the fraction *θ* of surface sites occupied by the adsorbed molecules to the concentration of adsorbate A at equilibrium, [A]_e_. If the surface density of adsorption sites is *Q*, the surface coverage Γ (in units of mass/area) of an adsorbate of mass *m_a_* is:

(4) $$\Gamma =m_{a}Q\theta$$

At the lowest level of complexity, the adsorption takes place as monolayer binding at sites are equivalent without any lateral interaction between the adsorbed molecules according to the Langmuir’s adsorption isotherm [19]:

(5) $$\theta =\frac{{\left[A\right]}_{e}}{\frac{1}{K_{L}}+{\left[A\right]}_{e}}$$

A real solid surface is, of course, generally characterized by different adsorption energies and therefore different Langmuir’s constants. For such an inhomogeneous system the overall adsorption isotherm is obtained integrating Equation (5) over all the *K_L_* values. It has been shown [20], that for a suitable distribution of adsorption energy, the result is what is called Sips [20] or the Langmuir–Freundlich equation [21]:

(6) $$\theta =\frac{{\left({\left[A\right]}_{e}\right)}^{n}}{\frac{1}{K}+{\left({\left[A\right]}_{e}\right)}^{n}}$$

where *K* is an average *K_L_*. In addition, for Equation (6), the constraint that the adsorbent can make only a monolayer as can be observed holds. The above equation is formally equivalent to the Hill’s model for the cooperative binding of a ligand to a receptor with *n* binding sites [22] but in the case of adsorption onto a surface the heterogeneity index *n* ≤ 1.

Accordingly, the surface coverage of Figure 2A was fitted to Equations (4) and (6).

**Figure 3:**
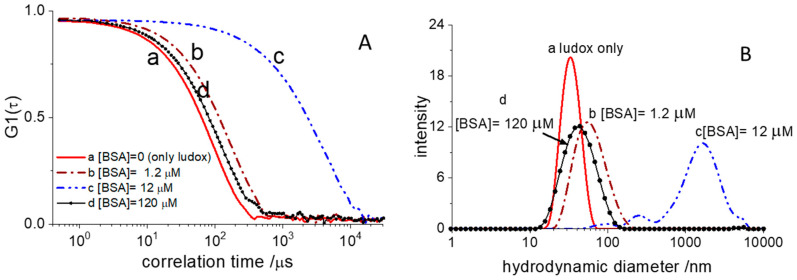
Effect of increasing BSA concentrations (0, 1.2, 12, and 120 μM) on a sample with constant Ludox concentration (2.5 mg/mL) in a 10 mM phosphate buffer (pH 8) and constant 25 °C measured by DLS. (**A**) Autocorrelation functions (ACF) and (**B**) intensity-size distributions of the experiments.

An analogous set of experiments was performed in solution by means of DLS using colloidal silica particles (Ludox) at a concentration of 5 mg/mL. DLS measures time-dependent fluctuations in the light scattering intensity, arising from particles undergoing Brownian motion in solution. At this concentration Ludox scatters almost ten times more than the highest BSA tested concentration and therefore DLS essentially probes the diffusional properties of the Ludox particles. The average sizes collected at all BSA concentrations are shown in Figure 2B, where the data can be easily compared with the adsorption isotherm measured on a macroscopic glass slide. Figure 3A shows representative autocorrelation functions (ACF) of the scattered light collected in these experiments.

The ACF of pristine Ludox (sample A) decays to zero at shorter correlation time than those measured in the presence of BSA. This behavior is an indication of BSA adsorption on the surface of the silica particles, thus increasing their hydrodynamic size. The most straightforward way to grasp such an evolution of dimensions is in terms of the size intensity distribution. This is the fraction of light scattered by particles of different hydrodynamic diameters and can be retrieved from the ACF, according to well-assessed numerical methods. The corresponding intensity distribution functions are shown in Figure 3B. The pristine Ludox particles are characterized by a narrow size distribution centered around 30 nm. Upon loading the sample with 1.2 µM BSA, the distribution moves towards larger sizes and becomes broader. For the sake of readability in Figure 3, we have shown only DLS experiments collected at four BSA concentrations. Initial addition of BSA triggers a dramatic increase in the hydrodynamic size that reaches micrometric values for BSA concentrations of 12 µM (Figure 3B) at which we expect half-saturation of the glass surface. At this concentration, the size distribution is extremely broad and multimodal. Overall, the DLS data collected at BSA = 12 µM suggests the presence of a large cluster population made by aggregated silica beads. Further BSA addition, however, leads to a decrease in cluster size that, at BSA = 120 µM, is only slightly larger than the original particles. 

A further indication of the presence of bead aggregates at intermediate concentrations comes from the intensity of the scattered light. The normalized scattering intensity can be calculated as the ratio between scattered intensity and the one collected for pristine non-aggregates Ludox. Assuming that most of the scattered light is due to the silica particles, whose concentration is constant throughout the experiment, and neglecting intraparticle interference, we can suppose the intensity of the scattered light is proportional to the aggregate’s molecular weight. Therefore, the normalized scattering intensity gives an estimate of the number of particles in the aggregates. These values are plotted in Figure 2B (right abscissa) and are parallel to the trend observed for the hydrodynamic size. At 120 µM BSA the normalized scattering is larger than 10 meaning that the micron-sized aggregates are made by more than 10 Ludox particles. The high affinity of BSA for macroscopic silica surface (Figure 2A) when exerted in solution of colloidal glass, accounts for the bell-shaped dependence of the aggregation with the BSA concentration observed in the experiments of Figure 2B.

When BSA concentration is low, the available protein is not enough to saturate the silica surface and there are many free binding sites on the silica nanoparticles. At BSA concentrations close to K^−1^, there are roughly the same amount of free and occupied binding sites. These are the conditions for a dramatic growth of three-dimensional clusters (akin to a gel formation). Experimentally, such a condition is fulfilled at a BSA concentration of 12 µM where, indeed, stable µm-sized aggregates form. The absence of macroscopic sedimentation suggests that, because of steric hindrance, a protein can bind only two nanoparticles and once the proteins have all been engaged there is no further aggregation. Increasing the BSA concentration, the system moves along the adsorption isotherm towards the surface saturation and the probability that an already bound BSA molecule will encounter another nanoparticle with an empty binding site decreases. When there is enough BSA to saturate the silica surface, the size of the aggregates decreases until only single silica nanoparticle coated by BSA is present (see Figure 2B for a pictorial representation) 

It is interesting to note how we find a monodisperse size distribution in the presence of BSA 120 µM with a mean size matching that of a Ludox sphere (30 nm) covered with a monolayer of protein (7 nm × 2 = 14 nm, 30 nm + 14 nm = 44 nm). Further confirmation comes from the electrophoretic mobilities, that are −2.43 ± 0.04 µm⋅cm/sV for pristine Ludox and −0.84 ± 0.11 µm⋅cm/sV for Ludox in the presence of BSA 120 µM. Assuming the particles are spheres, one can calculate the corresponding ζ-potential according to the Smoluchowski approximation to obtain −31.0 ± 0.5 for pristine Ludox and −11 ± 1 mV Ludox in the presence of BSA 120 µM. The latter value coincides with the ζ-potential measured for BSA in the same buffer and, therefore, strongly supports the scenario that under these conditions the system is formed by glass beads coated by BSA and thus represents the colloidal counterpart of the macroscopic silica surface covered by 450 ng/cm^2^ of BSA.

### 3.2. Surfactant Interactions with BSA-Coated Silica

**Figure 4:**
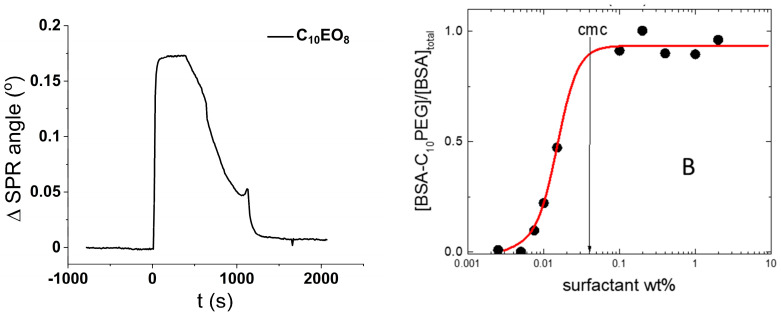
(**A**) SPR sensogram of the interaction of C_10_PEG with a SiO_2_ surface and (**B**) fraction of protein bound to surfactant in solution versus surfactant concentration calculated from fluorescence experiments as reported by Mateos et al. [17].

The surfactant subject of this study is a decanol ethoxylated with an average number of eight ethoxy groups on the hydrophilic moiety (hereafter denoted as C_10_PEG) that is widely used in detergent formulations. With respect to the interactions of other conventional surfactants relevant for industry, C_10_PEG has a negligible affinity for the glass surface as demonstrated in Figure 4A, which shows the SPR response to the exposure of a silica surface to a C_10_PEG solution (1 wt%). The initial jump after the injection of the surfactant solution is a mere transient, due to the high refractive index of the solution, and rinsing with water returns the very same SPR signal of the pristine silica. This means that the surfactant does not adsorb on the silica surface.

On the other hand, the interaction of C_10_PEG with BSA in solution is very mild and limited to the binding of few surfactant molecules to the protein without reaching denaturation [17]. A representative binding isotherm is shown in Figure 4B where the fraction of protein bound to surfactant is plotted against the surfactant concentration (the details of these fluorescence experiments are reported elsewhere [17]). It is clear from Figure 4B that the binding involves monomeric surfactants. Indeed, it starts well below the critical micelle concentration (CMC) and saturates just above the CMC.

**Figure 5:**
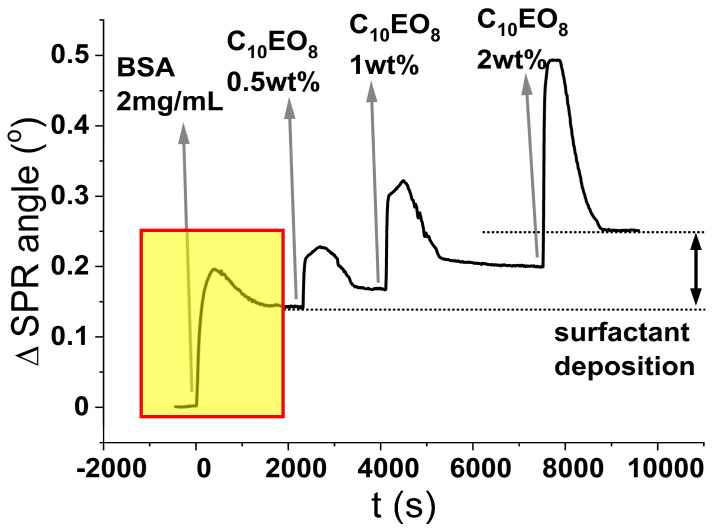
SPR sensogram of a kinetic titration for the adsorption of surfactant onto a pre-treated layer of BSA (highlighted in the yellow box).

In light of these premises, the results of the experiments shown in Figure 5 are unexpected. As seen from the isotherm presented in Figure 2A, the surface coverage of SiO_2_ by BSA reaches a plateau after injection of 30 μM BSA (= 2 mg/mL). We, therefore, used this concentration to pre-treat a new sensor surface onto which, we further injected increasing concentrations of surfactant. In this set of experiments, performed on the macroscopic planar surface of a SPR sensor, the silica surface was first exposed at a 30 µM BSA solution, a condition that leads to the formation of a stable protein layer as demonstrated by the stable plateau (0.15°) in the SPR response after rinsing (this takes place in the boxed region of the sensogram in Figure 5). After this, we flushed solutions of surfactant at increasing concentrations (0.5, 1, and 2 wt%) over the BSA-coated glass surface. Flushing with C_10_PEG results in a sequential increase of the SPR signal of sequential plateaus, which denotes an interaction at the sensor’s surface between surfactant and protein. 

**Figure 6:**
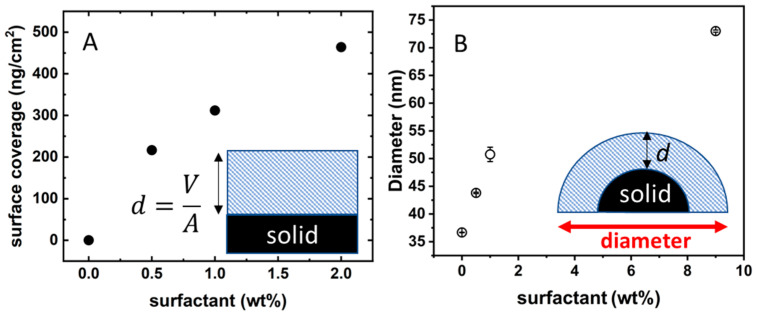
Increase in surface coverage of a flat surface measured with SPR (**A**) and hydrodynamic diameter of a colloidal surface measured by DLS (**B**) upon increasing surfactant concentration on previously BSA-coated SiO_2_.

It is possible to transform the SPR degree-signal into surface coverage as described in the previous section. The isotherm in Figure 6A shows the surface coverage (expressed as ng of dry surfactant per cm^2^) as a function of surfactant concentration. There are two very peculiar features in Figure 6A. First, when the BSA-coated silica surface is in contact with a 2 wt% C_10_PEG solution, the adsorption seems to be far from the saturation in marked contrast with the situation found for bare silica where there is no surfactant adsorption (Figure 4A). The second point is related to the values of surface coverage. The highest measured datum is 460 ng/cm^2^; taking as average molecular weight of 511 g/mol (of pure octaethylene glycol monododecyl ether) this corresponds to an average area per surfactant molecule of 18 Å. This is definitely too low for an area occupied by a surfactant with a bulky poly-ethoxylated moiety [23]. A possible explanation of both these features is that the surfactant is arranged in the form of multilayers.

To check such a hypothesis, we moved to analogous experiments performed on colloidal silica where we could easily follow the change in size upon addition of surfactant. The Ludox beads were first exposed to BSA 120 µM in order to have well-dispersed nanoparticles fully coated by proteins (diameter 39 ± 5 nm). The BSA-coated Ludox solution was then loaded with increasing amounts of C_10_PEG. The presence of surfactant induces a continuous increase in the hydrodynamic size of the particles, as shown in Figure 6B.

The increase in size is not as huge as expected in the case of particle–particle coagulation (see for example, Figure 2B) but it is however large. The lowest C_10_PEG addition is able to increase the hydrodynamic diameter by 7 nm which is the size of a C_10_PEG micelle. Loading with 9 wt% surfactant almost doubles the hydrodynamic size of the particles (remember BSA-coated Ludox has a size around 39 ± 5 nm and the increase at 9 wt% is of 36 nm). An increase of 36 nm in the hydrodynamic diameter indicates the adsorption of a layer of 16 nm, much larger than what one expects for the formation of a surfactant monolayer (~3.5 nm). Therefore, also the experiments performed on colloidal glass coated with BSA points towards the multilayer adsorption of C_10_PEG over the protein coating.

Of course, a quantitative comparison between the results obtained by SPR on flat surfaces and DLS on colloidal surfaces is very interesting. To have a fair comparison, we must take into account that DLS measures a hydrodynamic thickness that refers to a curved surface and that includes bound water. On the other hand, the surface coverage evaluated by SPR refers to a planar surface and is expressed in terms of dry mass of surfactant.

Here we propose to compare the results from these two experimental approaches in terms of effective thickness of a planar surface by correcting the SPR results for the bound water and the DLS data for the curvature as detailed in the following.

**Figure 7:**
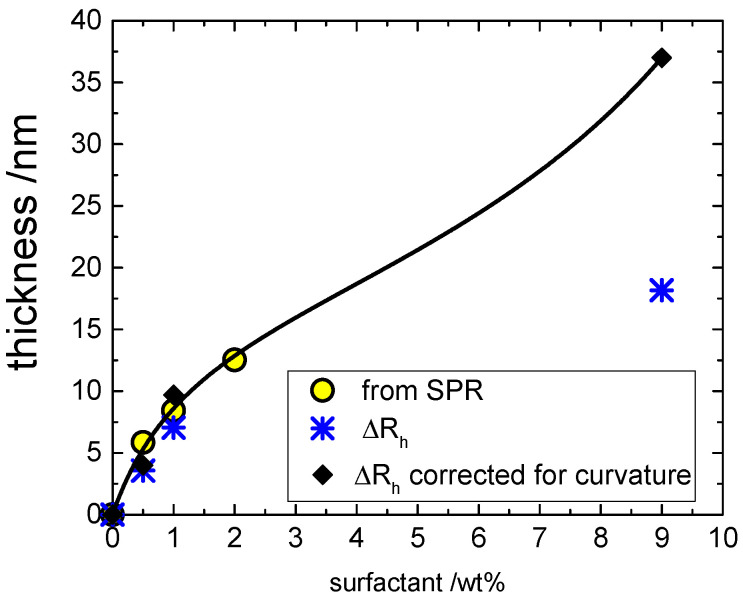
Comparison of the deposited layer of surfactant on the flat and colloidal BSA-coated SiO_2_ surfaces where the yellow circles are the values of “wet” thickness obtained by SPR on a flat surface, the asterisks are the hydrodynamic thickness obtained by DLS on a colloidal surface, the black diamonds are the hydrodynamic thickness values corrected for the curvature, and the black line is the fitting of the data using a BET isotherm (Equation (9)); best-fit values are *q*_1_ = 17 ± 3 nm, *K*_L_ = 0.8 ± 0.3, and *K*_ml_ = 0.062 ± 0.01 (these constants have dimensions of reciprocal concentration in wt%).

(1) SPR. Several papers point towards an average value of six water molecules associated to each EO group of non-ionic surfactants [24,25,26]. Having an average number of eight ethoxy group per surfactant molecule gives 48 hydration water molecules per polar head. Thus, one mol of C_10_PEG (511 cm^3^ assuming a density close to one) is associated to 864 cm^3^ of water giving an effective molar volume of 1375 cm^3^/mol. The surface coverage allows to evaluate the volume (water + surfactant) of the film deposited above the BSA-coated silica. For a planar surface, the volume-to-area ratio (V/A) equals the thickness of the film (assumed to be incompressible and homogeneous) as sketched in the inset of Figure 6A. The corresponding values of thickness of the hydrated film are shown in Figure 7 as yellow circles. Of course, such a calculation assumes the EO hydration in the micelles is not very different from the one found on the surfactant film. The validity of such an assumption was tested comparing the film thickness with the observed changes in the hydrodynamic size of glass beads (point 2 below).

(2) DLS. Figure 7 also depicts the hydrodynamic thickness (Δ*r_h_* asterisks) obtained by the DLS as the difference between the hydrodynamic radius in the presence of surfactant (*r*_h_) and the hydrodynamic radius of the original BSA-coated silica nanoparticle (*r*°_h_); in formula: Δ*r*_h_ = *r*_h_ – *r*°_h_. The two sets of data are in reasonable agreement, demonstrating that the correction of SPR data for hydration is consistent. However, the Δ*r*_h_ values are systematically lower than the corresponding film thickness coming from SPR. This is because the thickness of a curved layer underestimates the corresponding volume. For example, the volume of a spherical coating *V* = (4π/3)[(*R* + *d*)^3^ – *R*^3^] is larger than the product area × thickness = 4πR^2^d. For a film characterized by a mean and Gaussian curvature (*H*_c_ and *K*_c_, respectively), it can be demonstrated that the relation between V/A and the thickness *d* is [27,28]:

(7) $$\frac{V}{A}=d\left(1+H_{c}d+\frac{K_{c}d^{2}}{3}\right)$$

where *H_c_* and *K*_c_ are evaluated at the surface A. For a sphere of radius *R* the following equalities hold: *H*_c_ = *R*^−1^ and *K*_c_ = *R*^−2^ and we refer to the surface of the BSA-coated silica bead with a radius *R* ~ 20 nm so that it is possible to evaluate the corresponding thickness of a planar surface inserting the hydrodynamic thickness in the above equation obtaining

(8) $$\frac{V}{A}=d\left(1+\frac{\Delta r_{h}}{R}+\frac{\Delta r_{h}^{2}}{3R^{2}}\right)$$

The corresponding values of thickness for planar surface are shown in Figure 7 as black diamonds. It is clear that not only are they are in good agreement with the SPR results within the shared range of concentrations by the two approaches, but the DLS results obtained at high concentration clearly do not have any saturation. Instead, the overall isotherm has the classical shape of a type II adsorption isotherm [29] and accordingly it is accounted for by a Brunauer–Emmett–Teller (BET) isotherm [30]:

(9) $$\Gamma =\frac{q_{1}K_{L}\left[A\right]}{\left(1-K_{ml}\left[A\right]\right)\left(1+\left(K_{L}-K_{ml}\right)\left[A\right]\right)}$$

where the surface coverage is now expressed as volume of adsorbed hydrated surfactant per unit area (*d* = *V*/*A* nm^3^/nm^2^ in Figure 7), *K*_L_ is the affinity constant for the first monolayer (equivalent to the Langmuir’s constant), q_1_ is the corresponding saturation coverage, and *K*_ml_ is the affinity constant of the multilayers. The best-fit parameters are listed in the caption of Figure 7.

The data obtained by means of SPR and DLS indicate a strong affinity of the non-ionic surfactant for the BSA-coated surfaces that becomes an effective adsorption at concentrations well above the CMC. This contrasts with the experiments performed in solution (in the absence of any glass surface) where an interaction between surfactant and proteins takes place only at low concentration (below the CMC) as shown in Figure 4B. A weak point of the fluorescence experiments of Figure 4B is that they probe the protein (i.e., the saturation indicates that from the protein point of view the conformational changes induced by the surfactant binding have levelled off). In principle, it could be possible that the surfactant continues to adsorb on the protein. To check such a possibility, we have studied the surfactant/BSA interactions by means of diffusion-NMR (dNMR) [30]. This is a technique that allows the measurement of the surfactant self-diffusion coefficient (for a recent review on the applications of this technique to binding process see [31]). The rationale of the experiment is straightforward: by dNMR one measures the self-diffusion coefficient of the surfactant alone and in the presence of BSA without any interference from the protein (they have different NMR peaks). If a non-negligible fraction of the surfactant is bound to the slow-diffusing protein the observed diffusion coefficient will be reduced with respect to that of a micellar solution without BSA. For example, the binding of a cationic surfactant to BSA results in a 30% decrease in the surfactant diffusion and further elaboration of this evidence allows the quantification of the amount of bound surfactant [17].

In the case of a solution of C_10_PEG 2 wt% we measured a diffusion coefficient of (1.00 ± 0.05) × 10^−10^ m^2^/s. Loading this solution with BSA up to 4 mg/mL (60 µM) does not change the value of the diffusion coefficient within the experimental error. Such evidence is fully understandable if the surfactant saturates the protein below the CMC so that the maximum fraction of bound C_10_PEG is 0.02% of the total. 

The markedly different affinity of C_10_PEG for BSA in solution and adsorbed on a solid surface is unexpected. We found two different studies in literature that are in line with our evidence. Fully atomistic molecular dynamics simulation indicates that, upon adsorption on silica, BSA does not unfold but, however, undergoes a change in orientation that exposed towards the water more hydrophobic regions (while the more hydrophilic ones interact with silica) [15]. In another study, the changes induced by the adsorption are so large that it was possible to evaluate experimentally differences in the Hansen solubility parameters of BSA when it is in aqueous solution and in the form of an adsorbed layer [32]. 

## 4. Conclusions

In the present work we propose a method for the comparison of molecular interactions occurring on two types of surface geometries: flat and highly curved colloidal nanoparticles. The interaction of surfactant C_10_PEG on a BSA-coated SiO_2_ both in flat and colloidal forms has been demonstrated to follow the same behavior. This comparison is possible through a combination of techniques, each dedicated to a specific geometry (SPR for flat and DLS for colloidal surface geometries), correcting the obtained data by the water contribution and geometrical factors. 

The comparison of the interactions between BSA and C_10_PEG both free in solution and when adsorbed on a surface has been shown to be different and not predictable. Regardless of the geometry, the results of both SPR and DLS reveal a strong affinity of C_10_PEG towards BSA-coated surfaces at concentrations above the surfactant’s CMC. When free in solution, however, the interaction starts at very low surfactant concentrations (well below the CMC). These findings indicate different conformations of the protein in both states.

These findings indicate different surfactant/BSA interactions for BSA in solution and adsorbed onto silica. According to recent molecular dynamics simulations [15], BSA adsorption on SiO_2_ does not require any substantial changes in the protein conformation. However, the same investigation showed that the adsorbed BSA exposed hydrophobic parts of the BSA to the solution. Such an arrangement sets up a very different condition for surfactant adsorption if compared to the binding of individual surfactant molecules to separate proteins in solution.

These results would confirm the capability of colloidal particles to be a model surface for cleaning interactions on hard surfaces. We have found the use of nano-sized colloidal surfaces to be comparable to that of flat-macroscopic surfaces as model systems to study dirt-removal applications. Using colloidal surfaces could present many potential advantages since colloids made in almost any kind of material used in hard surfaces are available at a low cost and DLS sample preparation is easier when compared to the main techniques used to investigate these systems in flat-macroscopic surfaces, namely, SPR and Quartz Crystal Microbalance with Dissipation (QCM-D).
